# How Should Oxygen Supplementation Be Guided by Pulse Oximetry in Children: Do We Know the Level?

**DOI:** 10.3389/fped.2016.00138

**Published:** 2017-01-27

**Authors:** Ross Langley, Steve Cunningham

**Affiliations:** ^1^Department of Respiratory and Sleep Medicine, Royal Hospital for Sick Children, Edinburgh, UK

**Keywords:** oxygen, lower respiratory tract infection, oxygen saturation, hypoxia, hypoxemia

## Abstract

Supplemental oxygen is one of the most commonly prescribed therapies to children in hospital, but one of the least studied therapeutics. This review considers oxygen from a range of perspectives; discovery and early use; estimation of oxygenation in the human body—both clinically and by medical device; the effects of illness on oxygen utilization; the cellular consequences of low oxygen; and finally, how clinical studies currently inform our approach to targeting supplementing oxygen in those with lower than normal oxygen saturation.

Oxygen really is the elixir of life. It is vital for ATP production at the mitochondrial powerhouse of cellular function, a lack of oxygen spells cell injury and death. No wonder clinicians are so cautious of low oxygen states. But how cautious do we need to be? Is our physiological system able to manage low oxygen challenges? And what should be considered low from a clinical perspective?

This review will consider our understanding of oxygen: its history, physiological principles, monitoring in our bodies, the effects of low oxygen at a cellular level, and how much clinical studies inform our understanding of risks and benefits of supplementing oxygen.

## Oxygen—A Brief History

In the late 18th century, the Swedish pharmacist Karl Scheele and the English theologian–chemist Joseph Priestley did a series of independent combustion experiments with mercuric oxide and potassium nitrate inadvertently discovering a new “air” (Experiments and Observations on Different Kinds of Air, Vol. 1–6, 1775, London). After refinements by Antoine Lavoisier in 1778, the gas was named oxyge’ne (meaning acid former). The first recorded medicinal use was 5 years later by the French physician Caillens who treated a young woman suffering from tuberculosis.

The medicinal use of oxygen became popular by the end of the 18th century supported by the “Pneumatic Institution.” Despite some medical studies throughout the 19th century (1) with advanced understanding of physical and chemical properties of oxygen (the Fick Principle in particular), it was not until 1917 when the Scottish physician/physiologist John Scott Haldane published a seminal paper on the proposed therapeutic administration of oxygen that sparked a revolution for the use of oxygen in medicine (2): continuous oxygen delivery was more beneficial than intermittent use. Though it would not be until the 1960s, his theory was proved correct (3). Recent novel approaches to oxygen delivery include high flow devices and non-invasive ventilation strategies.

## Physiological Principles of Oxygen

**Figure 1:**
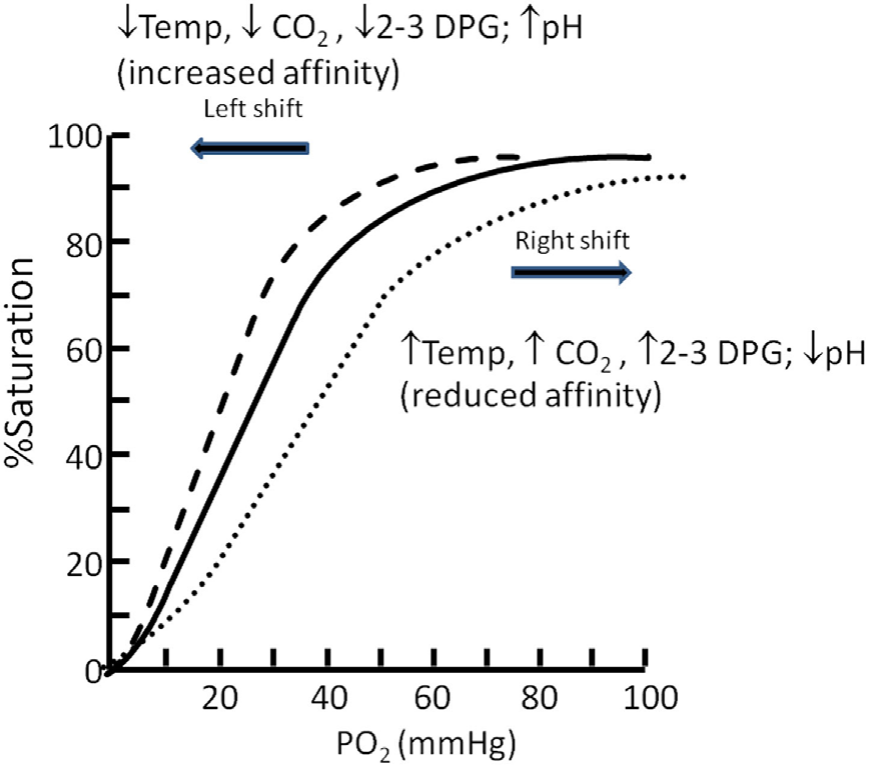
**Oxygen in children – what level**. Oxyhemoglobin dissociation curve.

Oxygen is carried in the blood principally bound to hemoglobin (Hb), but also dissolved within plasma. The relationship between oxygen saturation and blood oxygen content can be estimated with reference to the oxygen dissociation curve (Figure 1). This relationship is stable during health, but can be affected by a number of factors that influence the ease (or affinity) with which oxygen is bound to and released from Hb, including temperature, partial pressure of carbon dioxide, 2,3-diphosphoglycerate, and pH. A right shift makes it more difficult for oxygen to bind to Hb, but easier for it to be released at the tissues. A left shift has the opposite, easier binding but less readily released. As Hb becomes fully saturated, additional oxygen must be dissolved within plasma, which has a limited capacity under normal circumstances (but can be enhanced by supplementing oxygen above FiO_2_ 0.21). Decrease in pH/increased H+ ion shifts the curve to right and to the left with alkalosis. This is known as the Bohr effect. Carbon dioxide affects the curve in two ways: first, it influences intracellular pH (the Bohr effect), and second, CO_2_ accumulation generates high-level carb-amino compounds causing a right shift. 2,3-DPG is an organophosphate, generated in erythrocytes during glycolysis. The production of 2,3-DPG increases with respect to reduced peripheral tissue oxygen. High levels of 2,3-DPG shift the curve to the right. Low levels of 2,3-DPG is seen in states such as septic shock and hypophosphataemia and cause left shift. Hyperthermia causes a right shift, while hypothermia causes a left shift. Carbon monoxide binds Hb 240 times more readily than oxygen, and therefore interferes with Hb’s acquisition of oxygen. It lowers the potential for Hb to bind to oxygen and shifts the curve to the left *via* formation of carboxyhaemaglobin. In the context of an increased level of carbon monoxide, a person can suffer from severe tissue hypoxia while maintaining normoxaemia/normal PaO_2_ (4). Methemoglobinemia (a form of abnormal Hb) causes a left shift in the curve. Fetal hemoglobin (HbF) is structurally different from adult Hb. The fetal dissociation curve is shifted to the left relative to adults. Typically, the fetal arterial partial pressure of oxygen is low and hence the left shift enhances placental uptake of oxygen.

Normal values of oxygen saturation have been reported widely (5, 6) and at sea level (101 kPa/760 mmHg) oxygen saturation is within the normal range when reading between 94–100%. An oxygen saturation below 94% is hypoxemia. Oxygen saturations are lower in those living at altitude (estimated at a reduction of 5% per 1000 m altitude) (7). Once above an altitude of 6500 m, oxygen saturation stabilize at about 60–65% and do not drop further due to an increase in ventilation and the subsequent respiratory alkalosis shifting the oxyhemoglobin dissociation curve to the left (8).

## Assessing Oxygen

### Clinical Determination of Low Oxygen States

Historically, and today in resource poor regions, low oxygen status is determined clinically by identifying cyanosis – a dark blue/purple discoloration of the skin or mucous membranes (typically the tongue or inner lip). Cyanosis is one of the earliest and critical signs a medical student will be taught (9). It is one of the cardinal signs of severe illness, and failure to recognize cyanosis can appropriately lead to increased patient morbidity (10). Cyanosis is therefore considered a critical test of clinical ability to be able to appropriately identify and, if present, treat with supplemental oxygen. It is, however, a difficult clinical sign to elicit visually and recognition can be complicated by a number of factors including room lighting, skin pigmentation, and anemia.

For most clinicians, the point at which they are confidently able to discriminate someone as demonstrating cyanosis equates to approximately 85% oxygen saturation. However, detection of clinical cyanosis in respiratory illness varies appreciably from clinician to clinician (11), and identifying hypoxemia is significantly improved by availability of oxygen saturation measurement (12).

### Determination of Low Oxygen Using Medical Device

Arterial blood sampling for determination of arterial oxygenation is painful and invasive, and as a consequence of limited use in pediatrics outside intensive care. As a consequence, non-invasive surrogates for arterial oxygen, i.e., pulse oximetry, are extremely helpful in estimating oxygen saturation. Oxygen saturation monitoring is a relatively new technology, and although ubiquitous in developed healthcare settings, it is not available to the majority of people who require an estimate of oxygenation during ill health in developing healthcare settings (13). The principle of pulse oximetry is based on the red and infrared (IR) light absorption of oxygenated and deoxygenated Hb. Oxygenated Hb absorbs more IR light (wavelength 850–1000 nm) whereas deoxygenated/reduced Hb absorbs more red light (wave length 600–750 nm). Red (R) and IR light are emitted through an area of skin with good blood flow. A photodetector is sited opposite to the emitter and receives the wavelength of light passing through the measuring site. Light is increasingly absorbed during a pulse “wave” and more accurately reflects arterial blood oxygen levels. The R/IR ratio is calculated following photodetection. Typically, an R/IR ratio of 0.5 equates to approximately 100% SpO_2_, a ratio of 1.0 equates to approximately 82% SpO_2_, while a ratio of 2.0 equates to 0% SpO_2_. Measured values will be inaccurate if there is movement or extraneous light (particularly with incorrect sized clip in children) or if nail polish is *in situ*. Skin pigmentation should not affect accuracy. Oxygen saturation readings <70% are generally considered unreliable, as the monitors algorithms are imprecise below this level. Likewise, to improve accuracy, beat to beat measurements are not generally accepted due to artifactual influences; therefore, the SpO_2_ value represents an average overtime (a period typically of 10–15 s).

The introduction of pulse oximetry in a widespread manner in the 1980s and 1990s provided a clearly presented, precise number that staff find authoritatively acceptable. The step difference from our previous understanding of clinically detected cyanosis (85% oxygen saturation) to achieving normoxia (94% oxygen saturation) has provided a whole new level of clinical concern – what exactly does an oxygen saturation in the range 85–94% mean in the context of disease. Is it necessary to have normal oxygen saturation during illness?

Before the widespread use of oxygen saturation monitoring, supplemental oxygen was used either blindly (at a set level for a set time) or to relieve clinical cyanosis (i.e., oxygen saturation below c85%). The widespread availability of supplemental oxygen and precise, reliable, ubiquitous methods of estimating arterial oxygen provide clinicians with a capability for meticulousness that requires supporting evidence for benefits and harm.

## What is Low Oxygen?

Hypoxia is low tissue oxygen associated with tissue injury, whereas hypoxemia is low blood oxygen levels that may or may not be associated with hypoxia. The level of oxygen carried in the blood depends on the amount of oxygen bound to Hb and, to a lesser extent, dissolved in blood. In healthy humans breathing room air (21% oxygen) at sea level (101 kPa/760 mmHg), the partial pressure of arterial blood oxygen will be in the range 11–13 kPa (75–100 mmHg). At this level, intracellular oxygen is 2.7 kPa (20 mmHg) and mitochondrial oxygen is above 1.3 kPa (9.7 mmHg).

**Figure 2:**
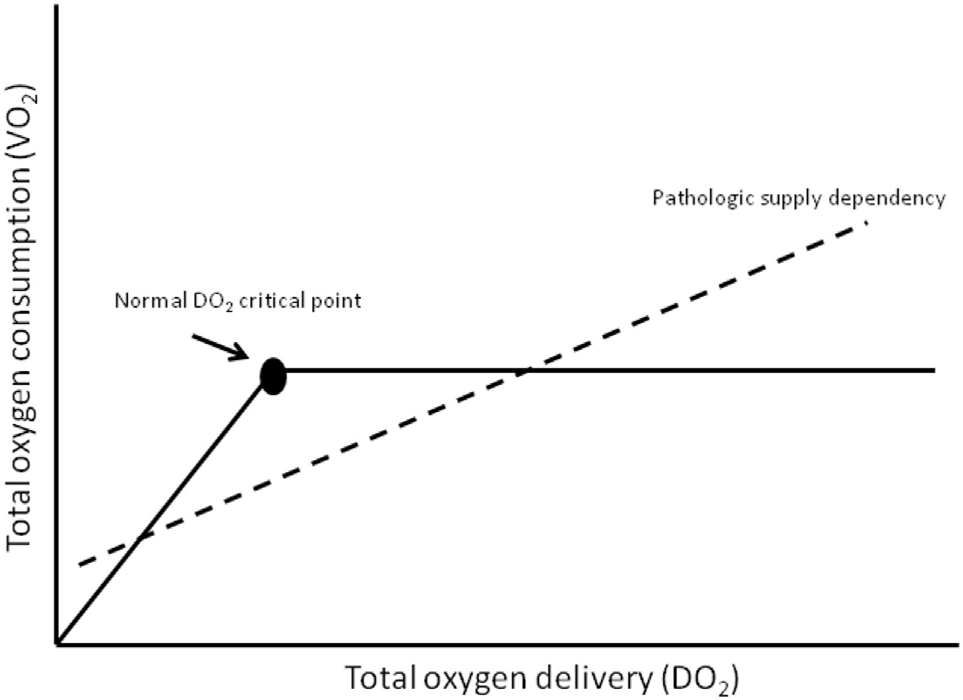
**Oxygen in children what level**. Adapted from Gutierrez and Theodorou (15).

Tissues cannot store oxygen and therefore must be supplied with a constant and steady supply. The global amount of oxygen delivery per minute is termed DO_2_. Reduction in DO_2_ is associated with reduced renal blood flow (with consequent reduction in urine output), lethargy, metabolic acidosis, and (if measured) a lower venous oxygen saturation (14). Delivery at a tissue level may be further compromised in those who are affected by anemia, hypovolemia, poor cardiac output, or edema of tissues. The global amount of oxygen consumed each minute is termed VO_2_. Global oxygen consumption (VO_2_) at a cellular level is increased by pyrexia, physical activity (increased respiratory rate or seizures), catabolic states, and pain, whereas VO_2_ is reduced by sedation, muscle paralysis, assisted ventilation and hypothermia. In normal health, oxygen consumption is supply independent; however, with illness, increased extraction and reduced delivery can compromise the DO_2_ critical point leading to supply failure and hypoxia (Figure 2).

Although global oxygen delivery and consumption can be determined, the ability to identify specific dynamic organ level risk is limited in day to day practice. Different organs and tissue groups have varied blood flow and oxygen uptake each minute, receiving varied amounts of cardiac output (Table 1). In hypoxic states, preferential shunting of blood away from tissues with low oxygen demand (skin, intestinal tract) help to maintain oxygen delivery to vital organs such as heart and brain which have high oxygen extraction rates and a poorer ability to tolerate low oxygen delivery. At a cellular level, reduced oxygen carried within capillaries reduces intracellular and consequently mitochondrial PaO_2_. Critical mitochondrial levels of oxygen lie in the range 0.01–0.10 kPa (16). Tissues particularly sensitive to low oxygen supply are renal tubular cells, cardiomyocytes, and neurons (17).

**Table 1:** **Oxygen in children – what level**.

Organ	% of Cardiac output	Blood flow (ml/min)	Oxygen uptake (ml/min)
Brain	14	840	52
Heart	5	300	34
Splanchnic bed	28	1,680	83
Kidney	23	1,380	19
Skeletal muscle	16	960	57
Skin	8	480	12

*Adapted from Ref. (18)*.

## Effects of Hypoxia on Cellular Level

Cells are vulnerable to both excessive and inadequate levels of oxygen. Protection from hypoxic/hyperoxic insults is achieved by complex homeostatic responses which influence cardiovascular, respiratory, and hematological parameters. The influence of hypoxia over the longer term on cardiac output (CO = HR × SV), ventilatory rate, as well as hematocrit and cellular oxygen demand/consumption may limit the negative influence of hypoxia. Ventilatory control is achieved *via* peripheral and central chemoreceptors. The effect of hypoxia on hematocrit is mediated *via* renal erythropoietin regulation.

**Figure 3:**
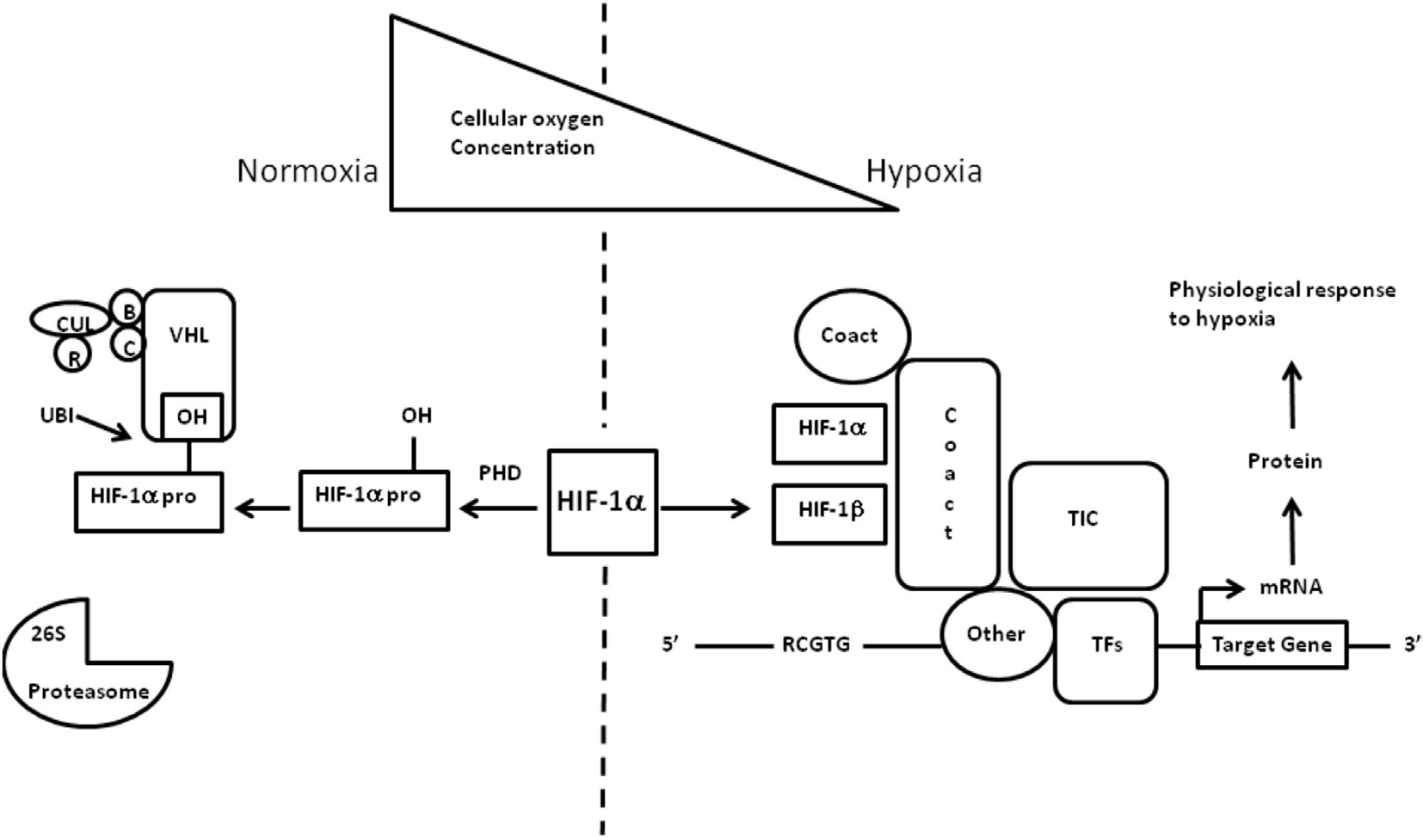
**Regulation of hypoxia-inducible factor (HIF)-1 oxygen in children – what level**. Regulation of HIF-1 adapted from Imtiyaz and Simon (19) and Semenza (20). HIF transcription factors are heterodimers consisting of an oxygen-regulated alpha chain bound to the constitutive beta subunit (aryl hydrocarbon receptor nuclear translocator). Under normoxic conditions, the alpha chain hydroxylated by the HIF prolyl hydroxylases leading to recognition by the von Hippel–Lindau (VHL) E3 ubiquitin ligase complex VHL which recruits elongins B and C, Cullin 2, and RBX1 (R) to constitute a functional E3 ubiquitin-protein ligase complex. Polyubiquitylation occurs and the alpha chain is degraded by the proteasome. Under hypoxic conditions, the HIF alpha chains are maintained and target gene transcription is enhanced. HIF-1 dimerizes and escapes prolyl hydroxylation, ubiquitination, and degradation. The HIF-1 heterodimer binds to hypoxia response elements containing recognition sequence 5_-RCGTG-3_ and recruits coactivator molecules resulting in increased transcription initiation complex formation. mRNA synthesis production and translation of proteins to mediate physiologic responses to hypoxia. TF = transcription factors.

**Figure 4:**
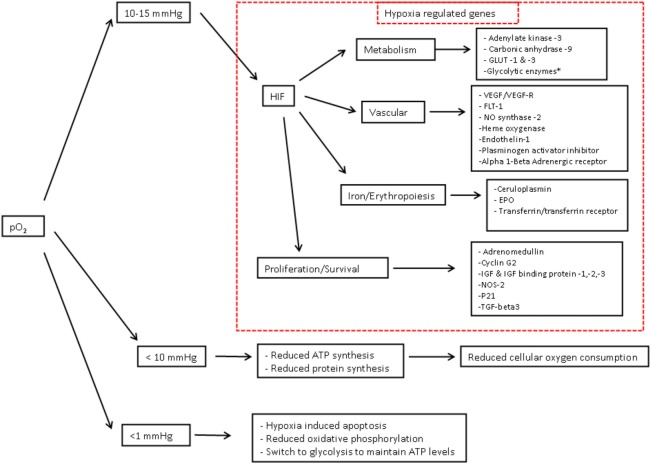
**Cellular consequences of hypoxia – Oxygen in children what level**. Cellular consequences of hypoxia—adapted from Semenza (20) and Span and Bussink (21). As partial pressure of cellular O2 falls, there are a number of target genes involved in oxygen homestasis. Hypoxia-inducible factor-1-regulated genes include the 11 glycolytic enzymes aldolase A, aldolase C, enolase1, glyceraldehyde-3-phosphate dehydrogenase, hexokinase 1, hexokinase 2, lactate dehydrogenase A, phosphofructokinase L, phosphoglycerate. kinase 1, pyruvate kinase M, and triosephosphate isomerase. Abbreviations: EPO, erythropoietin; HO, heme oxygenase; IGF, insulin-like growth factor; IGFBP, IGF binding protein; NOS, nitric oxide synthase; PAI, plasminogen activator inhibitor; TGF, transforming growth factor; VEGF, vascular endothelial growth factor. 1 mmHg = 0.13 kPa, 10 mmHg = 1.3 kPa, 15 mmHg = 2 kPa.

The cellular adaptive response to hypoxia is regulated *via* the hypoxia-inducible factor (HIF) family of transcription factors (19). HIF-1 is a heterodimeric protein comprising an oxygen-regulated subunit HIF-1 alpha and constitutively expressed HIF-1 beta subunit (aryl hydrocarbon receptor nuclear translocator) (20) (Figure 3). Under normoxic conditions, HIF-1 alpha is rapidly removed by polyubiquitylation and proteasome degradation. Under hypoxic conditions, specific enzyme activity is reduced, intracellular HIF-1 alpha accumulates and translocates to the nucleus. Subsequent dimerization with HIF-1 beta and binding to coactivators result in transcriptional activation of hypoxia-responsive genes involved in metabolism, cellular proliferation, vascular physiology, and erythropoiesis (Figure 4).

## Clinical Studies of Oxygen Therapy

The principle region of interest for studies of SpO_2_ in human clinical studies is the effect within normoxia (≥94%) and clinical cyanosis (85%). This region of interest oxygen saturation (85–94%) would not generally be considered symptomatic during health and could still be associated with adequate mitochondrial oxygenation, and so the benefits and harms of supplementing oxygen within this range during disease are of interest.

### Clinical Studies in Adults

There is physiological first principle evidence that low oxygen increases respiratory rate; however, supplemental oxygen as a therapy to provide symptomatic relief of dyspnea in adults is ineffective (22). A Cochrane review of oxygen supplementation in adults with respiratory disease demonstrates the lack of good quality evidence (23).

### Clinical Studies in Children

In children, the sensitivity of the developing brain to hypoxemia is much debated, yet has limited supporting evidence of benefits or risks in the region of interests (85–94% SpO_2_) (24). Hypoxemia is common in children who are unwell (25), in particular those with respiratory illness (26) and may often be undetected (27). The effects of hypoxia associated with newborn Hypoxic Ischemic Encephalopathy or out of hospital cardiac arrest are well described. Typically, such events are associated with a profound period of hypoxia often with marked acidosis and diminished cardiac output – though the ability of the young brain to withstand some episodes of prolonged hypoxemia can be impressive. Persisting hypoxia leads ultimately to cellular energy failure and cell death as the adapted mechanisms demonstrated above begin to fail. The boundary between hypoxemia and anoxia is termed dysoxia (28).

There is sufficient evidence (as above) to identify from physiological and biochemical principles that those who have cardiorespiratory or biochemical instability are most likely to be hypoxically challenged at a cellular level. Patients include those with significant acidosis associated with sepsis, dehydration, or diabetic ketoacidosis or those with a high probability of acute sudden reductions in global oxygen delivery, i.e., acute severe asthma or pneumothorax. In such patients, supplementing oxygen to normoxic values provides a reliable buffer against shifts in the oxygen dissociation curve or sudden challenges to oxygen delivery.

From a clinical perspective, the more challenging questions are based around the clinical management of patients who are biochemically stable (or with only mild pyrexia/increased carbon dioxide) and have hypoxemia within the range 85–94%, particularly over the longer term of days, weeks, or months. These include children recovering from acute lower respiratory tract infection or chronic lung disease.

### Chronic Oxygen Supplementation (Weeks or Months)

Preterm infants with lung disease often have hypoxemia and are provided with supplemental oxygen as a treatment. The early unrestricted use of oxygen for preterm infants in the 1950s was associated with a disastrous increase in the incidence of retinopathy of prematurity (29). The subsequent rebound restriction of supplemental oxygen reduced the incidence of ROP, but correspondingly increased death and brain injury (30, 31). In the 1970s and 1980s, the development and use of transcutaneous oxygen sensors enabled oxygen to be better titrated, controlling the risk of supplementing oxygen. Though ROP continued to be problematic even within transcutaneous oxygen target ranges (32). More recent studies in preterm infants have considered the risks and benefits of different target ranges for oxygen saturation. In a pre-planned international meta-analysis, a study was closed early when it became apparent that there were excess deaths in those infants managed within a lower oxygen saturation range (33). Of significant note was that the difference in the peak median oxygen saturation separating the two groups was just 3% SpO_2_; 89% in the lower and 92% in the higher range. In this vulnerable population of preterm infants, small differences in oxygen saturation over prolonged periods of time within the range interest (85–94% SpO_2_) has important effects on outcomes.

Children beyond the newborn period may also be considered a vulnerable population. The most studied population in this age group is children with obstructive sleep apnea (OSA). Children with reported OSA attain lower high school scores than contemporary peers without reported OSA (34, 35). When tested in a randomized controlled trial however, the anticipated effect on learning was not identified (36), creating further debate (37), though recent studies continue to suggest that prolonged repeated episode of airway obstruction (and possibly the associated hypoxia) do have cognitive effects in developing brains (37). Random repeated episodic obstructive events over many months result in both sleep architecture arousals and recurrent hypoxemic events; teasing out which is most associated with potential cognitive deficits is difficult.

### Acute Oxygen Supplementation in Respiratory Disease

Is it helpful to have a one size fits for oxygen saturation limits? Should our response to oxygen saturation be more responsive and intuitive to the patients’ illness and symptoms? As one of the most commonly prescribed treatments, oxygen supplementation and its effects on illness is poorly studied.

In general, there is very little evidence, of poor quality, supporting the use of oxygen in children with acute lower respiratory tract infection (38). Acute LRTI is the leading cause of mortality among children in developing countries and is responsible for up to 30% of all mortality in children under 5 years of age (39), with an estimated 120 million cases of pneumonia each year (40).

Hypoxemia is common in children with LRTI (41) and SpO_2_ <94% is present in 73% of children under one year of age admitted to hospital in a developed setting with acute bronchiolitis (42). In older children with pneumonia in a developing healthcare setting, hypoxemia (SpO_2_ <90%) is present in 13% (25). Outcomes are poorer in those with nutritional deficit at the time of hypoxia (43). The World Health Organization recommends an oxygen saturation target of 90% for supplementing oxygen in stable children (44). This is generally considered a pragmatic proposal in resource poor countries where oxygen provision is challenging, but also takes account of the stability of the oxygen dissociation curve at oxygen saturation greater than 90%. In high and middle income healthcare settings, oxygen is generally provided to maintain oxygen saturation above 92%.

In a pilot trial for children with pneumonia, early use of supplemental oxygen did not prevent subsequent development of hypoxaemial (45). Children with pneumonia, provided with supplemental oxygen to maintain SpO_2_ ≥90% had fewer deaths than those who did not receive supplemental oxygen (46).

There is just one randomized controlled trial of oxygen supplementation in acute respiratory disease in children. Bronchiolitis is the most common lower respiratory tract infection in children under one year of age. Most commonly caused by Respiratory Syncytial Virus the only effective management is maintenance of hydration and relief of hypoxemia. In 2005, a discrepancy arose between two guidelines for the management of bronchiolitis. With no evidence to support either position, a UK guideline recommending supplementing oxygen until normoxia was achieved (SpO_2_ ≥94%) and a US guideline recommended, on the basis of oxygen dissociation characteristics, that oxygen supplementation could be limited to <90% if the child was stable and improving. In a double-blind randomized controlled equivalence trial testing, these two oxygen saturation targets, outcomes for key clinical parameters were equivalent or possibly better for infants managed to the lower oxygen saturation target (42). Neurodevelopmental outcomes were not assessed within this trial, as the brief and improving oxygen saturation of trial subjects was not considered to be sufficient of neurodevelopmental concern—though this position is not accepted by all (47).

So do we know the correct target for oxygen saturation in acute lower respiratory tract infection in children? And what is it worth to know? In developed economies, there may be little appetite to push boundaries where outcomes are generally good and significant risk of severe neurodisability or death would be unacceptable outcomes (48). But just as early use of oxygen demonstrated harm as well as good, so possibly is the case with use of supplemental oxygen in children. In the Bronchiolitis of Infancy Discharge Study, outcomes appeared better in those managed to a lower oxygen saturation (and so fewer received supplemental oxygen for a shorter duration) with more speedy recovery (from a parent perspective) and a faster ability to regain feeding. Oxygen toxicity was postulated as possible for these effects (42). In developing healthcare, the ability to most effectively distribute scarce supplemental oxygen as a resource does support further evaluation of target oxygen saturation. These are proposed to be tested in an MRC trial in Africa (the COAST trial).

Do we know the level at which we should supplement oxygen? Not yet.

## Author Contributions

All authors listed have made substantial, direct, and intellectual contribution to the work and approved it for publication. Both authors revised the manuscript sections and completed editing.

## Conflict of Interest Statement

The authors declare that the research was conducted in the absence of any commercial or financial relationships that could be construed as a potential conflict of interest.
